# Prolactin receptor gene transcriptional control, regulatory modalities relevant to breast cancer resistance and invasiveness

**DOI:** 10.3389/fendo.2022.949396

**Published:** 2022-09-15

**Authors:** Raghuveer Kavarthapu, Maria L. Dufau

**Affiliations:** Section on Molecular Endocrinology, Division of Developmental Biology, Eunice Kennedy Shriver National Institute of Child Health and Human Development, National Institutes of Health, Bethesda, MD, United States

**Keywords:** prolactin receptor (PRLR), transcriptional regulation, gene structure, signal transduction, breast cancer, prolactin (PRL)

## Abstract

The prolactin receptor (PRLR) is a member of the lactogen/cytokine receptor family, which mediates multiple actions of prolactin (PRL). PRL is a major hormone in the proliferation/differentiation of breast epithelium that is essential for lactation. It is also involved in breast cancer development, tumor growth and chemoresistance. Human PRLR expression is controlled at the transcriptional level by multiple promoters. Each promoter directs transcription/expression of a specific non-coding exon 1, a common non-coding exon 2 and coding exons E3-11. The identification of exon 11 of PRLR led to finding of alternative spliced products and two novel short forms (SF) that can inhibit the long form (LF) of PRLR activity with relevance in physiological regulation and breast cancer. Homo and heterodimers of LF and SF are formed in the absence of PRL that acts as a conformational modifier. Heterodimerization of SF with LF is a major mechanism through which SF inhibits some signaling pathways originating at the LF. Biochemical/molecular modeling approaches demonstrated that the human PRLR conformation stabilized by extracellular intramolecular S−S bonds and several amino acids in the extracellular D1 domain of PRLR SF are required for its inhibitory actions on PRLR LF-mediated functions. Studies in breast cancer cells demonstrated that the transcription of PRLR was directed by the preferentially utilized PIII promoter, which lacks an estrogen responsive element. Complex formation of non-DNA bound ERα dimer with Sp1 and C/EBPβ dimers bound to their sites at the PRLR promoter is required for basal activity. Estradiol induces transcriptional activation/expression of the PRLR gene, and subsequent studies revealed the essential role of autocrine PRL released by breast cancer cells and CDK7 in estradiol-induced PRLR promoter activation and upregulation. Other studies revealed stimulation of the PRLR promoter activity and PRLR LF protein by PRL in the absence of estrogen *via* the STAT5/phospho-ERα activation loop. Additionally, EGF/ERBB1 can induce the transcription of PRLR independent of estrogen and prolactin. The various regulatory modalities contributing to the upregulation of PRLR provide options for the development of therapeutic approaches to mitigate its participation in breast cancer progression and resistance.

## Introduction

Prolactin (PRL) is a multifaceted protein hormone produced and secreted by the anterior pituitary gland, and it is also found in extra-pituitary tissues, including the mammary gland, brain, decidua, gonads, pancreas, immune cells, liver and adipose tissue, where it exerts autocrine and paracrine functions [reviewed in (1–4)]. Prolactin directs several physiological functions, such as lactation, immunomodulatory actions, and glucose and lipid metabolism, and is involved in pathological modalities, such as prolactinoma, hypogonadism and several cancers [reviewed in (2–4)]. The highly diversified actions of PRL are mediated through its transmembrane prolactin receptor (PRLR), a member of the lactogen/cytokine receptor family, which is expressed ubiquitously and functions as a dimer activated by PRL. A short isoform of PRLR with 310 amino acids (aa) was initially cloned from rat liver (5). The long form (LF) of PRLR with 610 aa was isolated from human hepatoma and breast cancer cells (6) and from the rat ovary (7). The monomeric structure of human PRLR (hPRLR) was resolved using combined NMR and computational approaches (8). PRLR gene expression is controlled by multiple promoters that regulate sustained PRLR levels and function (9). In humans, there are several PRLR isoforms, including the LF, many short forms (SFs), an intermediate variant and a soluble isoform [ (10), reviewed in (11)]. The expression of these isoforms of PRLR widely varies in different tissues and is required for specific functions of the organ system at specific times. Certain short isoforms of hPRLR can interfere with the essential signaling of the long isoform, thereby exerting inhibitory action [reviewed in (11, 12)].

PRL is structurally similar to growth hormone and can act as a growth factor, immunomodulator, or neurotransmitter in an autocrine and paracrine manner [reviewed in (2)]. PRL mediates its actions through PRLR, resulting in the activation of the janus tyrosine kinase (JAK)/signal transducer and activator of transcription (STAT) and mitogen-activated protein kinase (MAPK) signaling pathways [reviewed in (2)]. PRL/PRLR has been implicated in the development of several cancers and tumor progression [reviewed in (13–20)]. Hence, PRLR signaling has emerged as a relevant target in breast cancer. PRL is normally secreted in a pulsatile fashion, and this is clinically important for diagnostics related to problems in lactation and female infertility. Additionally, elevated circulating or locally produced PRL levels are associated with the risk of breast cancer [reviewed in (17, 18)]. PRL, through its cognate receptor, stimulates various downstream signaling cascades involving STAT5, RAS and MAPK, and phosphatidylinositol 3-kinase (PI3K) have been implicated in mammary tumorigenesis [reviewed in (18, 19, 21)]. The gain-of-function mutations in PRLR reported in benign and malignant breast cancer patients may support the hypothesis that PRLR signaling cascades could participate in benign breast tumorigenesis (22). In this review, we provide an overview of the current understanding of the transcriptional regulation of PRLR and signal transduction in physiological and pathological modalities in mammary glands with special emphasis on the role of PRL/PRLR signaling in breast cancer.

## PRL and PRLR

### PRL and PRLR distribution and biological functions

Prolactin is a 23 kDa protein hormone containing 199 aa that is produced in the lactotroph cells of the anterior pituitary gland. Prolactin is also secreted in an extra-pituitary and autocrine/paracrine manner from different tissues, such as mammary glands, brain, decidua, gonads, pancreas, immune cells, liver, and fat [reviewed in (1–3)]. The gene encoding human PRL is ∼10 kb with five exons, and four introns is present on chromosome 6. In addition to its normal expression in the anterior pituitary, it is also expressed in mammary epithelial cells, the decidua, brain, myometrium, endometrium, lacrimal gland, thymus, spleen, skin fibroblasts, sweat glands and immune system cells. PRL secretion is regulated by several factors. Ovarian steroids, specifically estrogens, modulate PRL synthesis and prolactin release while suppressing dopamine synthesis [reviewed in (1–3)]. Increased PRL secretion (prolactinomas) directly suppresses the secretion of GnRH and indirectly suppresses follicle-stimulating hormone and luteinizing hormone, thereby disrupting the HPG axis and the ovulatory cycle (reviewed in 1-2). PRL performs innumerable physiological functions in the body, including mammary gland development, lactation, gonadal functions (luteal cycle, uterine actions, Leydig cell development), parental behavior, preadipocyte differentiation, osmoregulation, angiogenesis, immunomodulatory function, islet cell proliferation, adrenal steroidogenesis, bone and calcium homeostasis, anovulation, reduced stress responses, oxytocin secretion and lipid metabolism. Prolactin also promotes neurogenesis in maternal and fetal brains [reviewed in (2, 3)].

PRLR has been identified in numerous cells and tissues of adult mammals. Cellular proliferation is also an important function of PRL in mammals. The expression of receptors (short and long forms) tends to vary with the stage of the estrous cycle, pregnancy, and lactation (23). The expression of PRLR in the late gestational fetal rat using *in situ* hybridization and immunocytochemistry showed that the long and short isoforms of PRLR were expressed during late fetal development (days 17.5 to 20.5). These studies showed PRLR transcripts were widely expressed in tissues from all three germ layers, in addition to the classic target organs of PRL (24). The expression of PRLR mRNA in the fetal adrenal cortex, gastrointestinal and bronchial mucosae, renal tubular epithelia, choroid plexus, thymus, liver, pancreas, and epidermis was higher than that in other tissues (23).

### PRLR structure and isoforms

PRLRs belong to the lactogen/cytokine receptor superfamily, which mediates the various cellular actions of PRL in different target tissues. PRL binds to preexisting PRLR dimers and acts as a conformational modifier, which results in the activation of the JAK/STAT pathway and MAPK and SRC kinases, thus leading to the induction of PRL responsive genes [reviewed in (4, 18)].

#### PRLR structure

PRLR has three main domains: extracellular, transmembrane and intracellular. The extracellular domain is divided into two fibronectin domains, D1 and D2, and the WS motif in D2 acts as a molecular switch during ligand-bound activation of PRLR (25). PRLR has a single-pass transmembrane chain, and the receptor chain does not possess kinase activity. The receptor chain is dependent on the associated kinases to transduce phosphorylation-based signal cascades. The intracellular domain includes proline-rich sequence-mediated JAK2 association to the prolactin receptor is required but not sufficient for signal transduction Box-1/2 sub-domains. Box-1 is known to interact with JAK2 and SRC family kinases such as FYN [reviewed in (4)]. The intracellular domain of PRLR-LF is intrinsically highly unstructured/disordered and binds to negatively charged lipids of the inner plasma membrane through conserved motifs resembling immuno receptor tyrosine-based activation motifs. However, this lipid association of the PRLR intracellular domain is not accompanied by induced folding and is independent of specific tyrosine phosphorylation. These attributes may contribute to regulating intracellular signaling (26). There are two short isoforms of hPRLR generated by alternative splicing to exon 11 (10).

#### PRLR gene and isoforms

**Figure 1:**
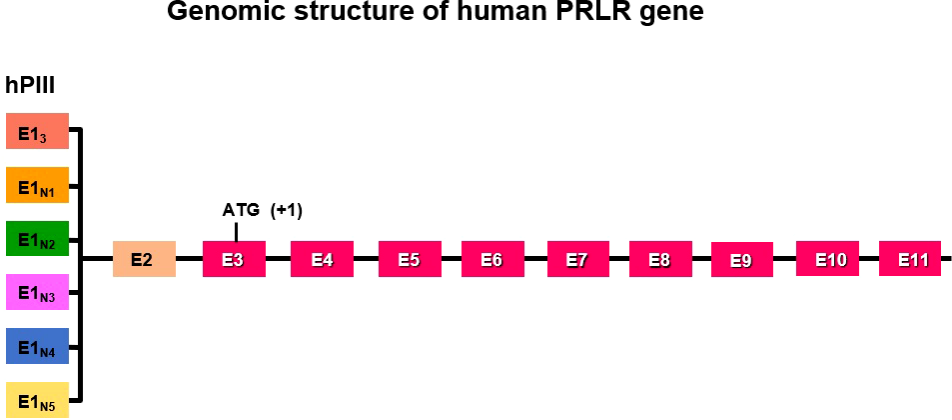
Schematic representation of multiple exons 1 (hE1_3,_ hE1_N1-5_) driven by individual promoters and alternative splicing to common exon 2 of human PRLR. Exon 3 has initiation codon. PIII/hEI3 is the predominantly utilized generic promoter in addition to five specific exon 1/promoters (hE1N1 to hE1N5).

**Figure 2:**
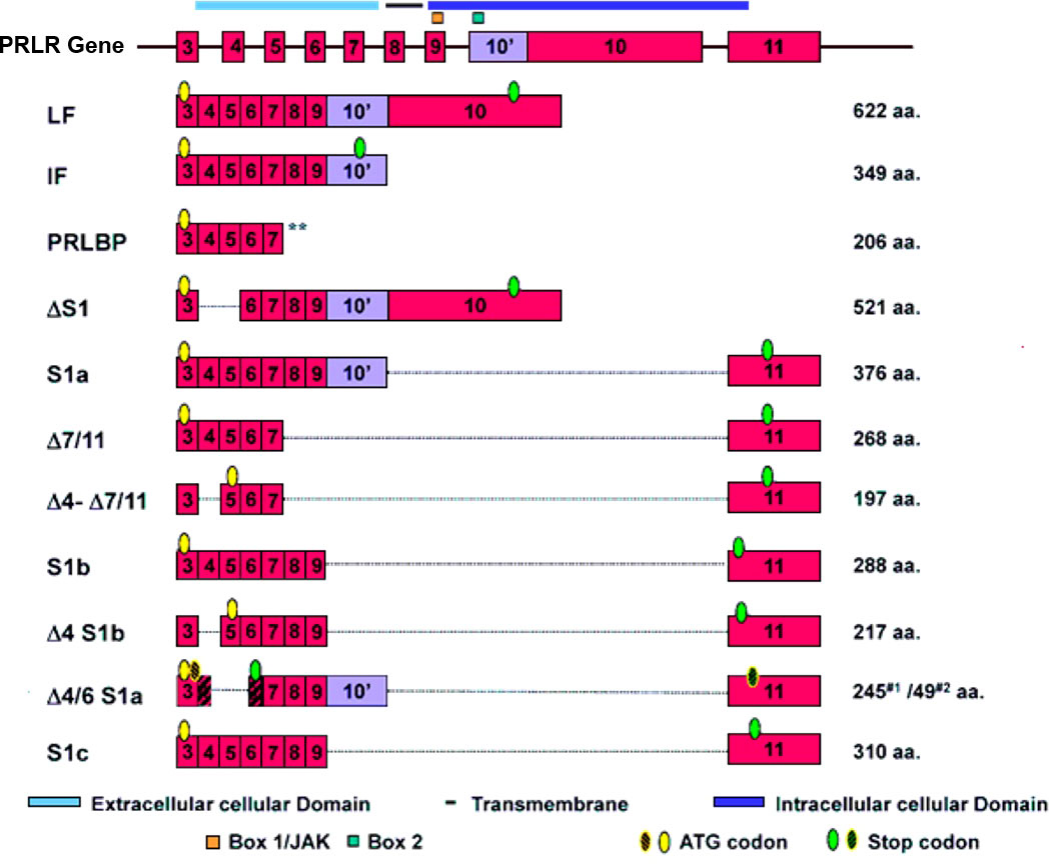
Schematic representation of human PRLR isoforms generated by alternative splicing [adapted from reference (27)] PRLR (prolactin receptor) Atlas Genet Cytogenet. ** indicates soluble form of PRLR.

**Figure 3:**
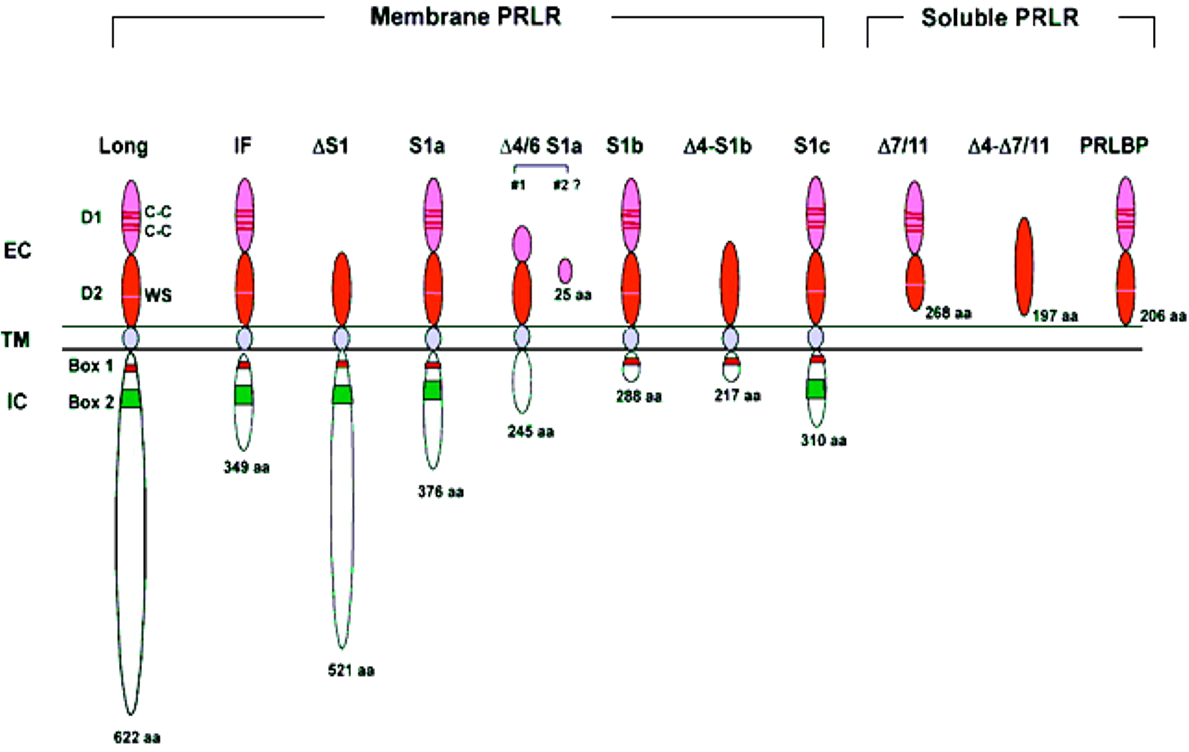
Structure of human PRLR variants. LF, long form; IF, intermediate form; S1a, S1b & S1c, short forms; PRLBP, prolactin binding protein; LFΔS1, long form lacking D1 domain; LFΔS2, long form lacking D2 domain; Δ, deleted exon. D1, D2, N-terminal subdomain; WS, WSXWS motif; C, cysteine; Y, tyrosine; EC, extracellular domain; TM, transmembrane domain; IC, intracellular domain [adapted from reference (27)].

The genomic organization of the hPRLR gene (>200 kb) is complex and subject to alternative splicing, which results in several isoforms of the receptor. The gene resides on chromosome 5p14-13. The hPRLR gene contains eleven exons, where exon 1 consists of six non-coding sequences (hE1_3_, hE1_N1-5_) that are alternatively spliced to a common non-coding exon 2, and only exons 3 to 11 are coding exons [ (9); **Figure 1**]. The LF of hPRLR is encoded by exons 3-10 (**Figure 2**). Additionally, an intermediate form (412 aa) with partial deletion of 198 aa within the cytoplasmic domain in exon 10 of the LF was isolated from the rat Nb2 lymphoma cell line (28) and human breast cancer cells (29). In addition, a soluble PRLR lacking transmembrane and cytoplasmic regions was isolated from the rat ovary (7). The transmembrane domain is encoded by Exon 8, while most of the intracellular domain is encoded by Exon 10 (**Figure 2**). Alternative splicing of exons 10 and 11 with a truncated intracellular domain resulted in two novel SFs of hPRLR, S1a and S1b, which inhibit the LF signal induced by PRL [ (10); **Figures 2**, **3**]. In addition, a unique spliced variant designated S1c, which completely lacks exon 10, has been identified in human spermatozoa (30). These short forms S1a and S1b are expressed as cell surface transmembrane receptors with a reduced cytoplasmic domain and unique C-termini S1b was far more effective in inhibiting the PRL-induced activation of the β-casein gene promoter mediated by LF (31, 32). A subsequent study demonstrated a naturally occurring ΔS2 deletion variant of SF in normal and cancerous human cells. These studies have also demonstrated that removal of the S2 extracellular subdomain can alter the conformation of the intracellular signaling region of the LF and both SFs (S1a and S1b), thereby supporting the concept that the conformation of the ECD can affect the conformation of the intracellular domain (33).

**Figure 4:**
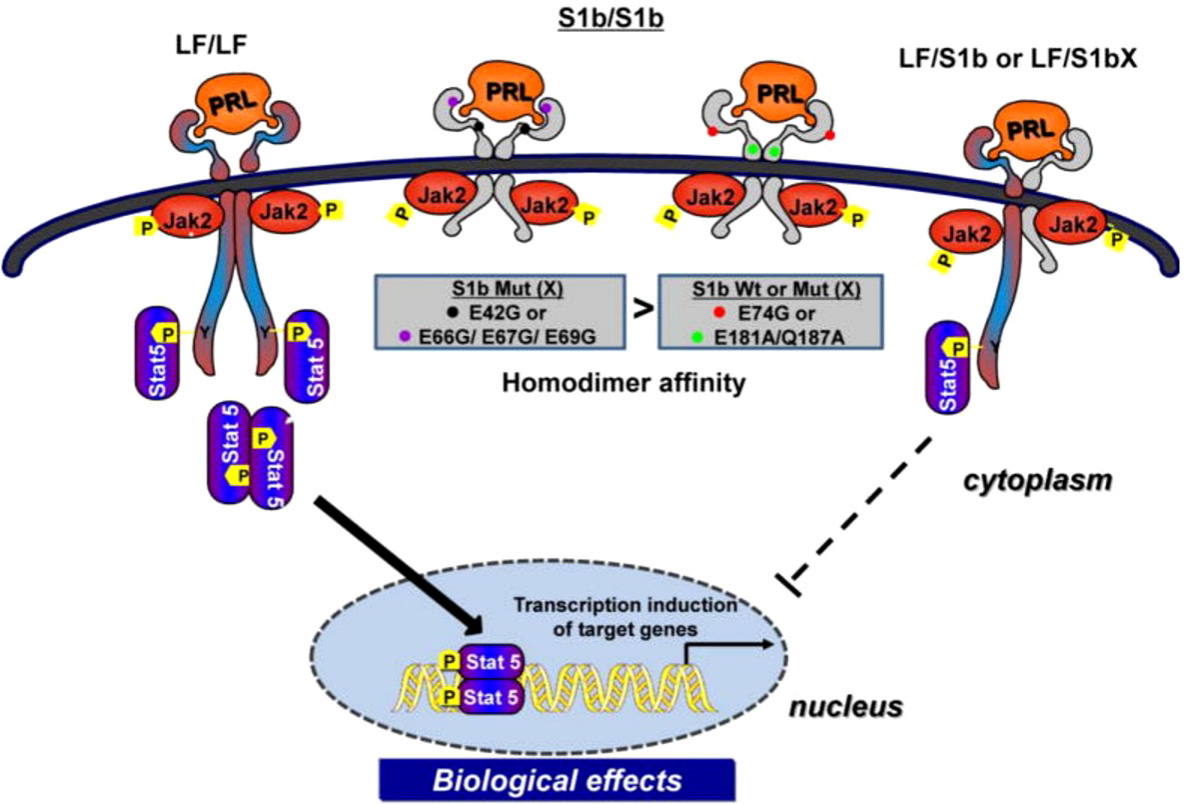
Mechanism of inhibitory action of short form S1b PRLR on PRL-induced long-from (LF) receptor signaling (reproduced from reference 12). Homodimer of the hPRLR long form (LF) mediates PRL stimulated JAK2/Stat5 signaling required for transcription/expression PRL/PRLR target genes which are essential for the various biological effects of the hormone. The inhibitory action of short form S1b on LF’s function induced by PRL results from LF/SF heterodimer formation and marked reduction of LF/LF homodimers which are required by Stat5 activation.

Our studies on human LF, S1a and S1b have revealed the existence of constitutive LF and SF homodimers and heterodimers (LF/S1a or LF/S1b) under non-reducing conditions in the absence of PRL that acts as conformational modifier (31). Both LF and SFs (S1a and S1b), as dimers, are capable of ligand binding and PRL-induced phosphorylation of JAK2, but only LF can activate downstream STAT5 signaling. S1a and S1b cannot induce the downstream activation of STAT5 due to the lack of an extended cytoplasmic domain. PRL signaling through the SF of PRLR in mouse ovaries actively regulates the expression of several genes and can profoundly affect follicular survival. SF can mediate the activation of MAPK and PI3K pathways [reviewed in (34)]. In breast cancer, the ratio of SFs (S1a and S1b) to LF is markedly reduced compared to that in adjacent tissue, which indicates that the loss of inhibitory regulation of LF could increase tumor cell proliferation (35). Two intramolecular disulfide bonds within the extracellular D1 domains are essential for the inhibitory function of S1b on LF. Additionally, the JAK2 association was disrupted. S−S bond disruption of S1b (S1bx) affects the dimerization interface, thus causing a significant decrease in LF heterodimerization with S1bx and an increase in homodimerization of S1bx. Therefore, stability of the PRLR structure by intramolecular S−S bonds is required for the inhibitory action of S1b on LF-mediated function (36). Additionally, mutations in E69 of the D1 domain of S1b and neighboring amino acid residues (R66, E67, E42) close to its surface binding domain cause a loss of its inhibitory effect, while those away from this region or mutants in the D2 domain have no effect. These findings underscore the significant role of extracellular D1 on the S1b conformation and its inhibitory action in PRL-induced LF function [ (12); **Figure 4**]. In addition, PRL signaling through SF of mouse PRLR can either stimulate or inhibit a substantial number of transcription factors in the decidua as well as ovary. Few transcription factors have been shown to be similarly regulated in both tissues, while most transcription factors are oppositely regulated by PRL (37). Additional studies are needed to better understand the role of alternatively spliced PRLR isoforms and the manner in which such splicing is regulated in breast cancer.

### Transcriptional regulation of PRLR

**Figure 5:**
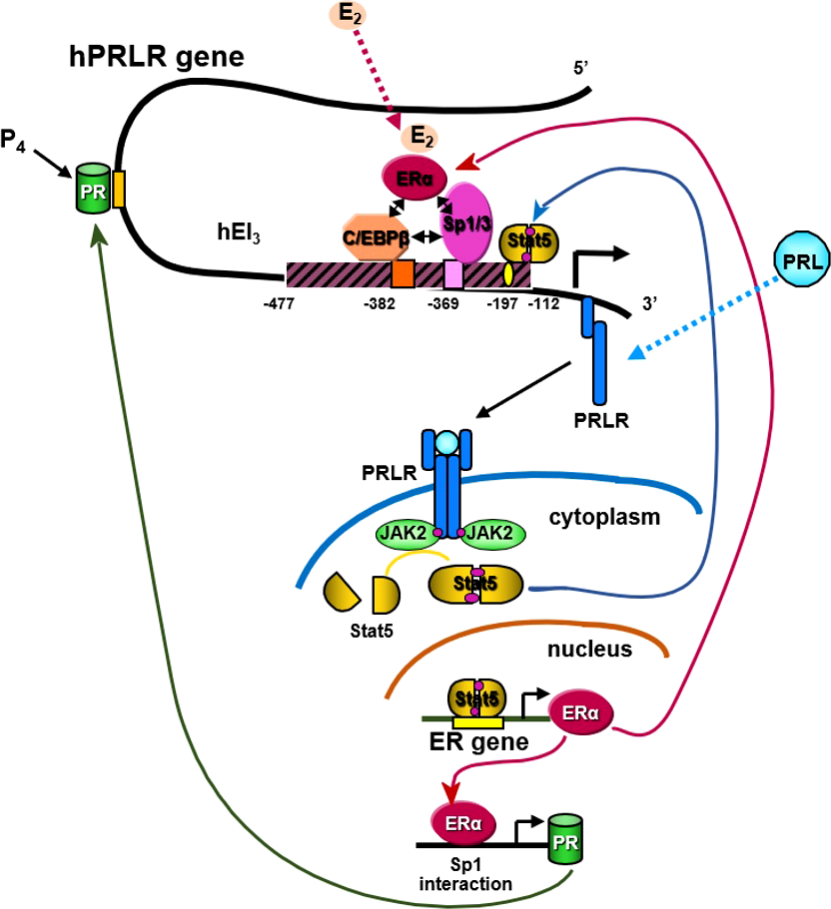
Transcriptional regulation of hPRLR induced by E2. E2 induces ligand mediated ERα-Sp1-C/EBPβ complex formation and binding to the hPIII promoter of the hPRLR gene (38, 39). This results in increased transcription of PRLR. PRL/PRLR activates JAK/STAT that can induce the transcription of ERα. P4 (progesterone)/Prog receptor can induce transcription of PRLR through association with Sp1and C/EBPβ through promoter hPIII.

The expression of PRLR is controlled by multiple promoters using a complex regulatory transcriptional network. In the rat these promoters are PI (gonad specific and SF1 dependent), PII (liver specific induced by HNF4), and PIII a widely expressed in all tissues and requires CCAAT/enhancer binding protein-β (C/EBPβ) and specificity protein 1 (Sp1) for its activation. Promoter PI is inoperative in mice due to disruption of the SF1 motif. These multiple promoters have been shown to direct tissue-specific expression in the ovary, liver and mammary gland in rats [ (38), reviewed in (39)]. In humans, hPIII is a universal/generic promoter similar in structure and regulation to one of those found in rodents (PIII). This promoter in humans directs transcription in all PRL-responsive tissues as well as other promoter(s) of less known function (9). The promoters include the predominantly utilized generic promoter 1/exon 1 (PIII/hEI3), which is also present in rats and mice, and five human-specific exon 1/promoters (hE1_N1_ to hE1_N5_) [ (9, 39); **Figure 1**]. The PRLR promoters belong to the TATA-less/initiator class and are activated by estradiol 17β (E2). The preferentially utilized human promoter III (hPIII) promoter contains Sp1 and C/EBP elements that bind to Sp1/Sp3 and C/EBPβ, which are required for basal transcriptional activity (**Figure 5**). These promoters were found to be utilized in breast cancer tissue and cell lines, including MCF7 and T47D, and variably used in other tissues (40). Among these promoters, the generic hPIII (the human counterpart of rodent PIII), which drives the universal human E1_3_ exon (the human counterpart of EIII in rodents), was functionally characterized in breast cancer cells, while the specific human exon hE_N1_ directed by promoter hP_N1_ is driven by domains containing an ETS element and a nuclear receptor NR half-site. The promoters for the specific human exons, i.e., hE_N2-5_, remain to be identified (39).

E2 can induce an increase in hPRLR mRNA transcripts directed by the hPIII promoter *via* a non-classical ERE independent mechanism in breast cancer cells [ (40); **Figure 5**]. The association of ERα with DNA-bound Sp1 and C/EBPβ is essential for E2-induced hPRLR gene transcription (**Figure 5**). The additional interaction between zinc fingers of Sp1 and leucine zipper of C/EBPβ stabilizes the ERα-Sp1-C/EBPβ complex. The enhanced complex formation of the ERα dimer (DNA binding domain) with Sp1 (zinc finger motifs) and C/EBPβ (basic region and leucine zipper) by E2 plays an essential role in the transcriptional activation of the PRLR gene [ (40, 41); **Figure 5**]. An autocrine/paracrine loop increases PRLR mRNA expression *via* its ligand PRL in breast cancer cells. Similarly, in MCF7 cells overexpressing PRL, upregulation of PRLR was observed in response to endogenous PRL but not exogenous PRL. Furthermore, there was an increase in ER levels and estrogen responsiveness in these MCF-7 cells. Owing to the positive effect of estrogen on PRLR transcription, this reciprocal regulation amplifies both ER and PRLR signaling in breast cancer (42). Another steroid hormone that can also regulate PRLR transcription is progesterone through its receptor (**Figure 5**). Progesterone participates in the menstrual cycle, pregnancy, and embryogenesis and can be involved in tumorigenesis as well as in normal growth. It has been reported that the progesterone receptor lacks the consensus sequence or half-sequence response element in the PRLR gene PIII promoter and demonstrated that progesterone induces an increase in PRLR mRNA in a non-classical manner by inducing the expression of PRLR through the cooperative activation of Sp1 and CEBPβ at the PIII promoter in mouse cells and T47D breast cancer cells (43).

## PRLR Signal Transduction

### JAK-STAT pathway

**Figure 6:**
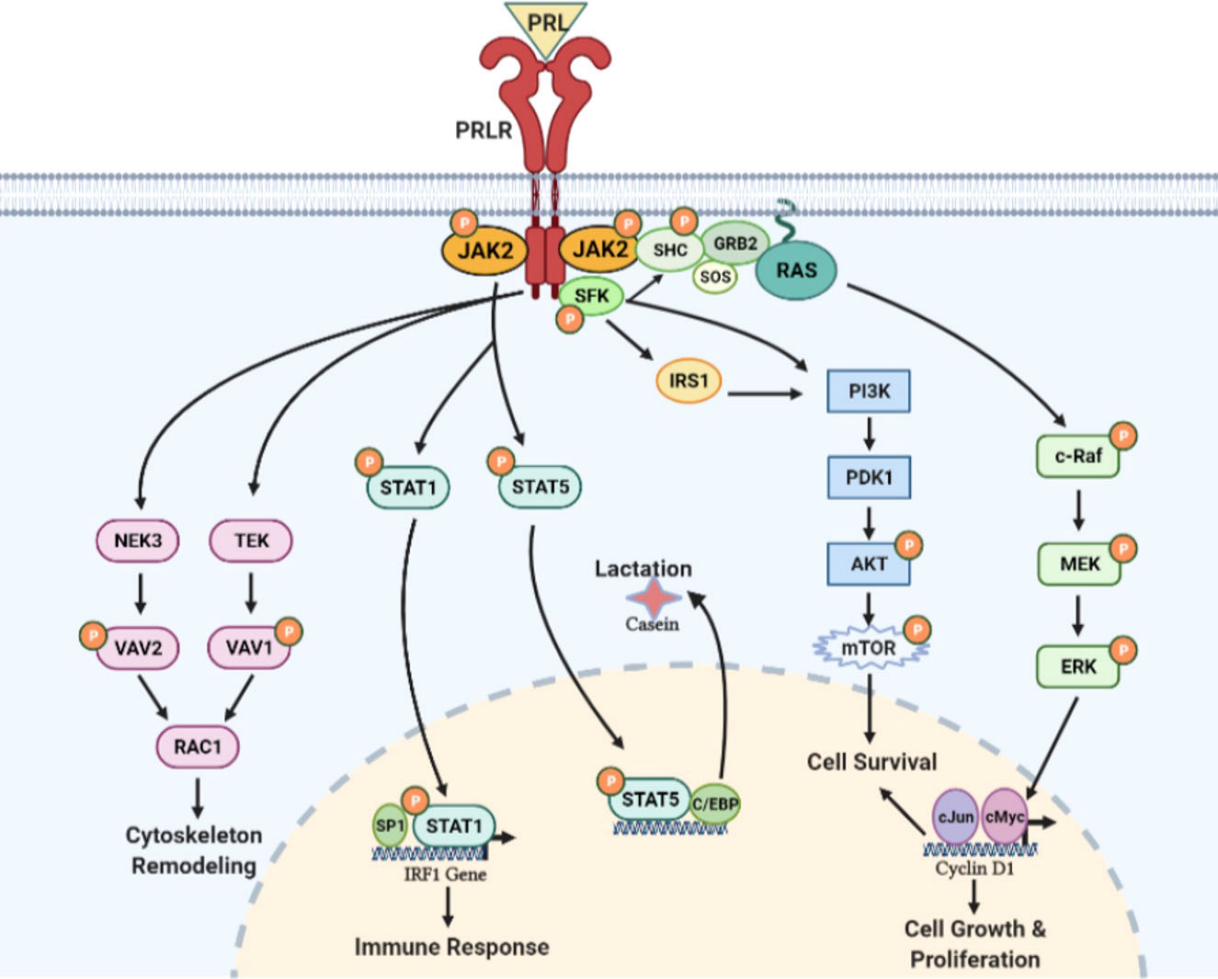
PRL/PRLR signaling pathways. PRL through its receptor PRLR induces multiple signaling cascades which include participation of JAK, SFK, PI3K/AKT, NEK3/VAV2, TEK/VAV1 kinases and STAT transcription factors. These signaling pathways are involved in lactation, immune response, cytoskeleton remodeling and cell growth, proliferation & survival [adapted from reference (44)].

This is the most classical and well-studied downstream signaling pathway induced by the binding of PRL to PRLR. This pathway appears to mediate most of the PRL actions in lobuloalveolar development and lactation (**Figure 6**). The intracellular domain of PRLR is devoid of any intrinsic enzymatic activity; however, ligand-mediated activation of PRLR results in tyrosine phosphorylation of numerous cellular proteins, including the receptor itself [reviewed in (2, 4)]. Binding of PRL to PRLR results in conformational induction of preformed dimers and activation of JAK2 by transphosphorylation, which brings two JAK2 molecules close to each other [reviewed in (2, 4)]. JAK2 kinases are involved in the phosphorylation of Tyr residues of the PRLR itself, and the phosphotyrosines serve as potential docking sites for transducer molecules containing SH2 domains [ (45), reviewed (4)]. The phosphorylation of Tyr residues of PRLR occurs in all isoforms except short isoforms of PRLR [reviewed in (4)]. The LF of PRLR mediates several processes upon receptor activation due to the phosphorylation of several Tyr residues present in PRLR (45).

The STAT family of proteins are the major transducers of cytokine receptor signaling, which contains eight members. STAT1/3 and STAT5a/5b have been identified as transducer molecules of PRLR. STAT contains five conserved domains: DNA-binding, SH3-like, SH2-like, and NH_2_- and COOH-terminal transactivating domains [ (46), reviewed in 4)]. As per the consensus model of STAT activation, a phosphorylated Tyr of the activated receptor interacts with the SH2 domain of STAT. Then, STAT is phosphorylated by receptor-associated JAK kinase. The phosphorylated STAT dissociates from the receptor and undergoes homodimerization or heterodimerization through an interaction involving the phosphotyrosine of each monomer and the SH2 domain of another phosphorylated STAT molecule. The STAT dimer translocates to the nucleus and activates a STAT DNA-binding motif in the promoter of target genes such as β-casein, IRF1, c-Myc, and cyclin-D (46). The consensus DNA motif (TTCxxxGAA), termed GAS (γ-interferon activated sequence), is recognized by STAT1, STAT3, and STAT5 homo or heterodimers [reviewed in (47, 48)].

### RAS/RAF/MAP kinase pathway

In addition to JAK/STAT signaling initiated by the activation of PRLR, several reports implicate PRLR in the activation of the mitogen-activated protein (MAP) kinase cascade (49, 50). Phosphotyrosine residues of PRLR can serve as docking sites for adapter proteins (Shc/Grb2/SOS) connecting the receptor to the RAS/RAF/MAPK cascade [ (49, 50); **Figure 6**]. Although the JAK/STAT and MAPK pathways were initially regarded as independent or parallel pathways, results suggest that these pathways are interconnected [reviewed in (51)].

### Other kinases: c-SRC and FYN

Several reports indicate PRL-induced activation of members of the Src kinase family, c-SRC (reviewed in 4) and FYN (52). PRL-induced rapid Tyr phosphorylation of insulin receptor substrate-1 (IRS-1) and a subunit of PI3K have been described. Both IRS-1 and PI3K seem to be associated with the PRLR complex. PRL-induced activation of PI3K has been proposed to be mediated by FYN (52). PRL/PRLR can also induce TEC-VAV1 and NEK3-VAV-1/VAV-2 signaling cascades and function in the regulation of the cytoskeleton (**Figure 6**). NEK3 kinase has been shown to regulates PRL-mediated cytoskeletal reorganization and motility of breast cancer cells (53, 54).

## Role of PRL/PRLR signaling in breast cancer

### PRL/PRLR induced signaling cascades promote breast development and progression

PRL plays a crucial role in mammary gland development and in the etiology and progression of breast cancer. Considerable supporting data indicate that PRL/PRLR hyper signaling contributes to the initiation of breast cancer. A strong correlation between breast cancer with increased PRL and PRLR has been reported in several studies (17, 55–58). Higher levels of PRL in postmenopausal women may eventually lead to an increased risk of breast tumors and metastatic cancer (58–60). In premenopausal women with breast cancer, there are higher-than-average levels of serum PRL together with elevated PRLR expression. These are associated with an increased risk of tumor progression and invasion (60–62). An association is observed between invasive breast cancer risk in postmenopausal women with high circulating PRL, particularly for ER-positive disease. PRL/PRLR is expressed in 95% of mammary tumors and 60% of male breast carcinomas (63). These findings were replicated in transgenic mice overexpressing PRL that develop mammary tumors and in *in vitro* studies where PRL played a role in the proliferation of breast cancer cells (64, 65). A direct correlation is observed between single nucleotide polymorphisms (SNPs) in the *PRLR* gene and benign breast tumor incidence. The two SNPs PRLR-I76V and I146L demonstrate constitutive receptor activity, and one of the SNPs (PRLRI146L) correlates with benign breast disease in a patient cohort, but these patients did not have high levels of serum PRL (22, 66). However, in subsequent study these SNPs in the PRLR gene were found not to be associated with breast cancer and multiple fibroadenoma (67). In another study, SNPs were found in *PRL*, and *PRLR* genes were associated with breast cancer metastasis in Taiwanese women (68). Studies from our lab and others in T47D and MCF-7 breast cancer cells have shown that the PI3K/AKT and RAF/MEK/ERK pathways are activated in parallel following PRL treatment, which leads to profound cell proliferation and survival (69, 70). PRLR can also induce the MAPK/ERK signaling cascade *via* the PI3-kinase-dependent RAC/PAK/RAF/MEK pathway, which is in turn controlled by JAK2, SRC family kinases and focal adhesion kinase (FAK) (71). In addition to the role of the predominant LF of PRLR in breast cancer, a recent study showed that the human intermediate PRLR (alternatively spliced isoform) is a mammary proto-oncogene capable of stimulating cell survival and proliferation (29). Many breast tumors are characterized by reduced STAT5 and high levels of PRLR expression and MAPK signal components, including AP-1 and pro-invasive matrix metalloproteinases [reviewed in (56)]. MMPs are highly invasive agents and are associated with resistance to chemotherapy and anti-estrogen treatments (72). Extracellular matrix components in the breast tumor microenvironment can also influence PRL/PRLR signaling (73, 74). In invasive breast cancer, there is a shift in PRL signaling from STAT5-mediated pathways to focal-adhesion kinase and MAPK pathways, thereby favoring proliferation (73, 74). PRL signaling in high-density stiff collagen matrices increases MMP expression, thereby promoting cellular motility. Therefore, the tumor microenvironment may be responsible for favoring one signaling pathway over another (73, 74). There is a complex interplay between PRLR and estrogen receptor (ERα), and there is an important role for the tumor microenvironment. The co-expression of PRLR and ERα in a non-compliant, rigid matrix is associated with increased tumor invasiveness and reduced responsiveness to estrogen antagonists (75). PRLR signaling can also induce motility and invasion of T47D breast cancer cells by activating downstream effectors such as TEC and NEK3 kinases, thus leading to cytoskeletal and focal adhesion reorganization (54, 76). Several studies using breast cancer cells have shown that PRL activates unliganded ERα through phosphorylation at the Ser118 and Ser167 residues. The activation of ERα promotes ligand-independent transcriptional initiation of ERE-dependent target genes, which seems to be an important factor in the proliferative and transcriptional actions of PRL in breast cancer cells (70, 77, 78). The most significant transcription factor in PRL/PRLR signaling is STAT, which regulates the growth, differentiation, and survival of mammary tissue. STAT3 and STAT5 are activated/overexpressed in several types of cancers, including breast cancer [reviewed in (79, 80)]. STAT5 can act as both a tumor suppressor and an oncogene in breast cancer under different circumstances. In ER-positive breast cancer, STAT5 expression enhanced the response to hormone therapy and increased the overall survival of patients [reviewed in (81)]. Recent studies have shown that phosphorylation of STAT5a serine residues (S726 and S780) may regulate its activity to promote cell proliferation in MCF-7 cells (82). Reports have indicated that STAT5 acts as a suppressor of breast cancer invasion and metastatic progression and can be used as a tumor marker of favorable prognosis [reviewed in (79)]. STAT5 is progressively inactivated with the progression to metastatic breast cancer due to enhanced regulation by tyrosine phosphatases (83). The activation of STAT5 in breast cancer cells promotes homotypic adhesion and inhibits the invasive characteristics of cells (84).

### PRL and cyclin-dependent kinase 7 (CDK7) in estrogen-induced upregulation of PRLR in breast cancer cells

We demonstrated the essential role of endogenous PRL in the upregulation of the PRLR promoter, which involves the requisite participation of E2/ERα at the hPIII promoter along with STAT5a (**Figure 5**). Phosphorylated STAT5a, which associates with its functional element at hPIII, interacts with non-DNA-bound E2/ERα, which in turn associates in a complex with Sp1 and C/EBPβ bound to their cognate DNA sites at the PRLR hPIII promoter [ (85); **Figure 5**]. We have shown in MCF-7 cells that E2 induces ERα phosphorylation at S118 *via* CDK7 kinase and greatly increases the recruitment of E2/ERα to the hPIII promoter over basal unliganded ERα (86). Phosphorylation of ERα at S118 is necessary for its association with the Sp1-C/EBPβ complex and its interaction with STAT5a. Inhibition by the specific CDK7 inhibitor THZ1 markedly reduced E2-induced ERα phosphorylation at S118, while the JAK2 inhibitor AG490 or MEK inhibitor U0219, which inhibits downstream JAK2-induced pathways known to phosphorylate unliganded ERα at S118 and S167, had no effect. Targeting CDK7 kinase, which is known to regulate both transcription and the cell cycle, and ERα phosphorylation with the THZ1 inhibitor was found to effectively inhibit the transcription of PRLR and cell migration in breast cancer cells (85). Our studies may provide insights for therapeutic approaches that will mitigate the transcription/expression of PRLR and its participation in breast cancer progression fueled by E2 and PRL *via* their cognate receptors (**Figure 5**).

### Crosstalk between PRLR and other receptors in breast cancer

**Figure 7:**
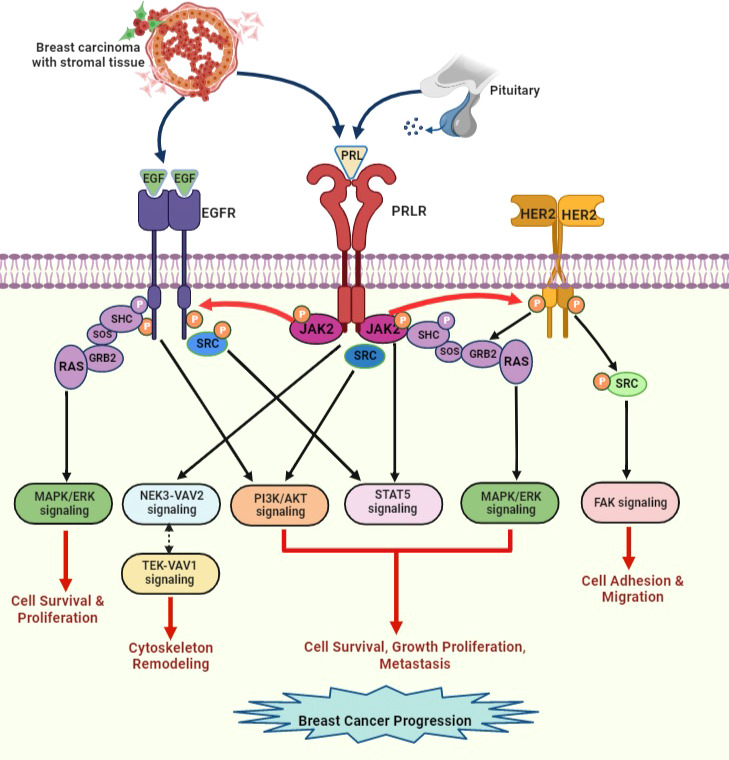
PRLR and EGFR/HER2 signaling crosstalk in breast cancer. EGF released by stromal microenvironment surrounding the breast tumor activates signaling cascades that overlap with PRLR signaling cascades upon activation with PRL secreted by breast tumor cells. PRL stimulates HER2 and EGFR signaling pathways via JAK2. EGF/EGFR also activates STAT5 signaling indirectly via s-SRC. This crosstalk between receptors can increase progression of breast tumor and endocrine resistance [adapted from reference (44)].

Studies have indicated that PRLR signaling crosstalk with other receptors can influence signal transduction. PRLR interacts with integrin *via* the signal regulatory protein alpha transmembrane glycoprotein and SHP2 (87). PRL/PRLR and E2/ER synergistically can regulate the gene expression and proliferation of breast cancer cells (87). Furthermore, PRLR signaling tends to activate the unliganded ER (70, 77). PRL and estrogen cooperatively induce phosphorylation of ERK1/2 and enhance prolonged activation of AP-1 in breast cancer cells (88). This type of signaling pathway crosstalk can promote breast cancer progression and chemotherapeutic resistance. Crosstalk occurs between PRLR and EGFR/HER2 (**Figure 7**). PRLR can activate HER2 signaling *via* JAK2 (70, 85). We have demonstrated that PRL/PRLR induces HER2 phosphorylation at Tyr residues 1221 and 1222 through JAK2, thereby activating downstream PI3K/AKT pathways in both MCF-7 and T47D cells (70). This crosstalk between PRLR and HER2 signaling further facilitates the phosphorylation of ERα, its recruitment to the PRLR promoter and upregulation of PRLR transcription. Interestingly, we also found that EGF/EGFR in MCF-7 and T47D cells can induce PRLR transcription *via* downstream MAPK and PI3K signaling pathways (**Figure 7**). Crosstalk between the PRLR and progesterone receptor (PR) signaling pathways has been shown to be relevant to both breast development and progression. Both PR and STAT5a are key transcription factors in these pathways and have been shown to be mediators of breast cancer stem cell outgrowth (89). This evidence, coupled with their established function in the same transcriptional complexes at phospho-PR-target genes with high cancer relevance, supports the importance of PR-PRLR crosstalk [reviewed in (90)].

### Resistance to endocrine therapy in breast cancer and future perspectives

Endocrine therapy is one of most effective forms of targeted adjuvant therapy for hormone receptor-positive breast cancer. Adjuvant therapy has been well established with different types of antiestrogens, including selective ER modulators (tamoxifen, raloxifene), which block the activity of ER, selective ER downregulators, such as fulvestrant, which causes destabilization and degradation of ER, and the third generation of aromatase inhibitors (anastrozole, letrozole and exemestane), which reduce the production of E2 in tumors [ (91), reviewed in (92)]. Although these endocrine therapies for women with ER+/PR+ breast cancer have led to substantial improvements, a significant number of cancer patients develop either intrinsic resistance or acquired resistance, which often results in tumor relapse. There can be multiple reasons for this endocrine resistance which includes mutations of ER, enhanced MAPK and PI3K/mTOR signaling pathways. Other aspects of endocrine resistance could result from overexpression of HER2, and crosstalk of ER with bypass signaling pathways, such as the EGFR/HER2 and PRLR signaling pathways [reviewed in (93–96)]. To overcome HER2 hyperactivation, trastuzumab is still being used as the most effective form of treatment for ER+ and HER+ breast cancer patients. However, some cancer patients develop resistance to trastuzumab and tumor relapse within one year of treatment. Hyperactivation of HER2-induced downstream PI3K/AKT signaling is often observed in trastuzumab-resistant breast cancer patients [reviewed in (97, 98)]. HER2-targeted therapies have been established in recent years, including tyrosine kinase inhibitors, such as lapatinib, neratinib, tucatinib, and pyrotinib (99). Together, these drugs targeting multiple receptors, such as HER2, EGFR and HER4, were studied in the early and advanced stages of breast cancer and revealed some promising outcomes. Furthermore, in clinical trials, the combination of HER family inhibitors with endocrine therapy has been shown to have better results [reviewed in (100)]. PRLR and EGFR/HER2 crosstalk, which greatly increases the activation of the RAS/ERK and PI3K/AKT pathways, are associated with poor prognosis and therapeutic resistance in breast tumor patients. In the case of PRLR, only a few attempts have successfully developed a potential therapeutic small molecule inhibitor or monoclonal antibody (LFA102) to block PRLR signaling induced cell proliferation in breast cancer cell lines (101). Therefore, simultaneous treatments targeting both the HER2 and PRLR signaling cascades may offer better outcomes by efficiently hindering breast tumor progression and ameliorating endocrine resistance. A study using G129R (PRLR antagonist) and trastuzumab (monoclonal antibody targeting HER2) as a combination therapy to inhibit HER2+ breast cancer cells and a nude mouse xenograft model showed inhibition of cell proliferation (102). Additionally, combining PI3K/AKT/mTOR pathway inhibitors with endocrine therapy has been shown to potentially reverse resistance to trastuzumab in HER2+ patients and metastatic breast cancer in early clinical trials. A rational combination of therapeutic agents based on the disease profile would be more beneficial to breast cancer patients [reviewed in (103)].

## Concluding remarks

PRL is a pleiotropic hormone that plays a crucial role in mammary gland development, lactogenesis, reproduction and immune function. It mediates its actions through PRLR, a member of the lactogen/cytokine receptor family. PRLR activation induces JAK/STAT and mitogen-activated protein kinase signaling pathways implicated in the development of mammary glands and etiology of breast cancer. In this review, we provide an overview of the current understanding of the complex organization of the human PRLR gene and its transcriptional regulation. Preclinical data, epidemiological studies, and patient tumor tissues analyses strongly support the contribution of PRL/PRLR to breast tumorigenesis and cancer progression. This review also stresses the importance of signal transduction pathways (PI3K/AKT, RAF/MEK/ERK, FAK, and SFK) activated by PRL/PRLR in breast cancer. We have summarized how steroid hormones (E2 and PR) and growth factors (EGF/ERBB1 and HER2) can induce the transcription of PRLR, thereby increasing its expression in breast cancer cells and promoting cell proliferation. Therefore, in this era of precision medicine, we conclude that combination therapy involving pathway-selective kinase inhibitors and PRLR inhibitors depending on the status of the breast cancer can provide better outcomes in clinical studies.

## Authors contributions

RK and MD were responsible for manuscript writing and editing. All authors contributed to the article and approved the submitted version.

## Funding

This work was supported by the National Institutes of Health Intramural Research Program through the Eunice Kennedy Shriver National Institute of Child Health and Human Development.

## Acknowledgments

The authors thank all laboratory members who participated in the studies covered by this review.

## Conflict of interest

The authors declare that the research was conducted in the absence of any commercial or financial relationships that could be construed as a potential conflict of interest.

## Publisher’s note

All claims expressed in this article are solely those of the authors and do not necessarily represent those of their affiliated organizations, or those of the publisher, the editors and the reviewers. Any product that may be evaluated in this article, or claim that may be made by its manufacturer, is not guaranteed or endorsed by the publisher.
